# Robust physical methods that enrich genomic regions identical by descent for linkage studies: confirmation of a locus for osteogenesis imperfecta

**DOI:** 10.1186/1471-2156-10-16

**Published:** 2009-03-30

**Authors:** Peter Brooks, Charles Marcaillou, Maud Vanpeene, Jean-Paul Saraiva, Daniel Stockholm, Stephan Francke, Reyna Favis, Nadine Cohen, Francis Rousseau, Frédéric Tores, Pierre Lindenbaum, Jörg Hager, Anne Philippi

**Affiliations:** 1 IntegraGen SA, 4 rue Pierre Fontaine, 91000 Evry, France; 2 Généthon, CNRS UMR8115, 1 rue de l'Internationale, Evry 91000, France; 3 Department of Pharmacogenomics, Johnson & Johnson PRD, PO Box 300, Raritan, New Jersey 08869, USA

## Abstract

**Background:**

The monogenic disease osteogenesis imperfecta (OI) is due to single mutations in either of the collagen genes ColA1 or ColA2, but within the same family a given mutation is accompanied by a wide range of disease severity. Although this phenotypic variability implies the existence of modifier gene variants, genome wide scanning of DNA from OI patients has not been reported. Promising genome wide marker-independent physical methods for identifying disease-related loci have lacked robustness for widespread applicability. Therefore we sought to improve these methods and demonstrate their performance to identify known and novel loci relevant to OI.

**Results:**

We have improved methods for enriching regions of identity-by-descent (IBD) shared between related, afflicted individuals. The extent of enrichment exceeds 10- to 50-fold for some loci. The efficiency of the new process is shown by confirmation of the identification of the Col1A2 locus in osteogenesis imperfecta patients from Amish families. Moreover the analysis revealed additional candidate linkage loci that may harbour modifier genes for OI; a locus on chromosome 1q includes COX-2, a gene implicated in osteogenesis.

**Conclusion:**

Technology for physical enrichment of IBD loci is now robust and applicable for finding genes for monogenic diseases and genes for complex diseases. The data support the further investigation of genetic loci other than collagen gene loci to identify genes affecting the clinical expression of osteogenesis imperfecta. The discrimination of IBD mapping will be enhanced when the IBD enrichment procedure is coupled with deep resequencing.

## Background

Mapping of regions identical-by-descent (IBD) is a powerful method for the identification of genetic loci shared within families and implicated in disease. Classically, typing of individual genetic markers throughout the genomes of the afflicted individuals mapped shared haplotypes and has been successful in finding loci linked with numerous monogenic traits [1]. An alternative physical method, Genomic Mismatch Scanning (GMS) [2], physically compares genomes of two affected individuals, related by a not too distant common ancestor, and enriches for the IBD regions they share. Despite its promise, the technique has not been exploited due to technical complexities.

As GMS offers the potential to avoid certain ambiguities associated with genotyping and may be applicable to pools of DNA samples from afflicted, related individuals, we aimed to improve the technique to reduce its inherent noise and to render it robust.

As compared with linkage discovery by genotyping, physical methods based on direct comparison of genomic sequences would enable more complete access to all IBD regions of the genome. Such methods rely on the fact that non-IBD regions are densely polymorphic between two individuals. A conceptually attractive approach for such a direct comparison involves the formation of duplex heterohybrid DNA fragments from the DNAs of related individuals sharing a trait of interest, and then challenging these fragments with reagents actuated by mispairings in the heterohybrids. Such reagents may bind to the mismatched fragment or may introduce strand breaks to permit separation of hybrid fragments that are not perfectly complementary from those that are perfectly paired from the IBD regions. Physical comparison has been successfully used with a variety of technologies that exploit the chemical or structural differences between perfectly matched and mismatched hybrids. These include chemical mismatch cleavage [3] and various attempts to harness proteins that respond to mismatches such as resolvase [4,5], single-stranded DNA-specific nucleases [6,7], MutS mismatch binding [8-10] or cleavage by the mismatch-specific enzymatic activity of E. coli MutS, MutL and MutH [11]. In addition, transfection of hybrid molecules into bacteria enables enrichment for perfectly paired fragments via an in vivo process dependent on mismatch repair activities [12,13].

The use of these mismatch recognition methods has generally been limited to the analysis of a few targeted fragments, but adapting them to genome-wide analysis is conceivable. Global treatment of fragments from the entire genome, however, also requires elimination of reannealed homohybrid fragments (fragments between strands of DNA from the same individual formed during hybridization, including both mismatch-bearing hybrids formed with one paternal and one maternal strand and isohybrids comprising strands from the same parent) whose presence would confound the identification of IBD DNA. Ford and colleagues[14,15], in presenting the concept of genome-wide enrichment of IBD fragments, introduced the strategy of tagging one genome with methyl groups, and then using restriction enzymes specific for either methylated or non-methylated DNA, but inactive with hemimethylated DNA, to remove homohybrid DNA. In addition, they suggested using various mismatch-specific agents, either by immunoprecipitation or by the E. coli mismatch repair system to eliminate mispaired fragments [12,14]. Subsequently, Nelson et al. [2] described GMS, a combination of the methylation-dependent homohybrid elimination method with in vitro cleavage by the mismatch repair proteins MutS, MutL and MutH, and digestion by exonuclease III. Application of GMS to pairs of related yeast strains, including mapping of the IBD-enriched regions by hybridization to arrayed clones, correctly identified meiotic recombination crossover points [2].

Use of GMS with mammalian genomes was demonstrated by enrichment of microsatellite alleles shared between related individuals [16,17] and confirmation of loci containing previously documented disease-related genes with mapping of IBD regions by hybridization to DNA arrays targeted to the chromosome of the known locus [18] or by microsatellite allele recovery [19]. Despite the recognition of the potential of GMS [20,21], the approach has not been widely exploited, due to a lack of availability of the mismatch repair proteins, various technical challenges inherent in a multi-step procedure and the lack of appropriate means to map the IBD-enriched DNA. A fundamental problem has been an apparent lack of appreciation of the need for a highly efficient process to ensure elimination of non-identical hybrid fragments. Such residual fragments will hybridize to the DNA features on microarrays and so increase non-specific noise. Modifications to GMS and IBD mapping have been reported but the results from genome wide studies showed a substantial lack of concordance to confirm CEPH family meiotic crossovers [22]. As linkage studies require processing of many samples, an additional limitation has been the use of reagent volumes and methods unsuitable to high throughput microtiter plate-based procedures.

We present here an improved protocol for physical enrichment of IBD regions. The monogenic disease osteogenesis imperfecta (OI; Brittle Bone Disease) provides a context for demonstrating that the protocol could correctly identify a well-established monogenic locus and an opportunity to discover novel loci relevant to OI etiology. OI includes a heterogeneous group of autosomal dominant inherited disorders characterized by bone fragility and other generalized connective tissue abnormalities [23]. As analysis of locus-specific restriction fragment length polymorphisms had shown that nearly all cases of OI segregated with the two collagen genes [24-28], genome-wide linkage analysis was not perceived as necessary. However, given the wide variety of clinical expression of OI, it is possible that variants of modifier genes may also cosegregate with OI [29]. Therefore a genome wide analysis might detect loci harbouring such genes.

We have implemented an IBD enrichment protocol that overcomes many of the difficulties of the original GMS protocol. We report that application of the improved protocol to DNA from OI patients has correctly identified the shared IBD locus that includes COL1A2 bearing the disease-causing mutation and additional loci that may be relevant to OI etiology. The protocol is now suitable for finding linkage loci in applications ranging from monogenic disease to complex multigenic disease.

## Results

### Overview of IBD enrichment process

**Figure 1:**
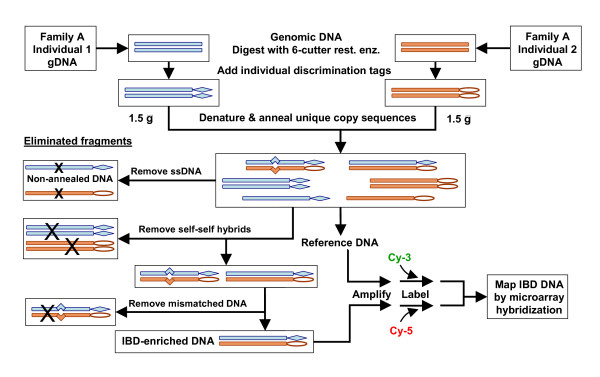
**Physical IBD enrichment process**. Genomic DNAs (gDNA) are isolated from two related, afflicted individuals and digested with a restriction enzyme that leaves Exonuclease III-resistant ends and generates fragments of about 4 Kb. DNA fragments are modified (diamonds or ovals) to permit discrimination between the individuals, for example by presence or absence of methylation at GATC sequences. The fragments are mixed, denatured and renatured under conditions to favour unique copy reannealing. Non-annealed strands and hybrids of strands from the same individual are eliminated. Reannealed DNA fragments with one strand from each individual are called heterohybrids, which may be perfectly paired due to inheritance of both strands from the same ancestral sequence or mismatched due to variation between different ancestral sequences. Mismatched heterohybrid fragments are removed by LSHase, a nucleolytic cocktail of MutL, MutS and MutH, and subsequent digestion by Exonuclease III. The resulting IBD-enriched DNA is generically amplified, labelled and mapped by two-colour hybridization to genomic topographic arrays, using the reannealed DNA as reference. The process is repeated for other afflicted pairs in the same family and in additional families. Variations include use of oligonucleotides as discrimination tags, reducing the number of steps by combining similar intermediate purification procedures, and eventually mapping IBD regions by high throughput redundant sequencing, with or without amplification.

The procedure for IBD enrichment can be applied to any related pair of individuals and is outlined in Figure 1. Affected relatives may have the same phenotype because they share a particular set of predisposing alleles inherited from common ancestral chromosomal regions. The procedure enriches for loci that, due to patterns of parental meiotic recombination events, are identical between the siblings, and thus include the predisposing alleles. The final IBD-enriched product is generically amplified and the IBD regions are then mapped by various methods, such as by hybridization to DNA microarrays or high throughput sequencing.

Genomic DNA (gDNA) from each individual is cleaved with restriction enzymes that yield fragments of sufficient length to ensure a high probability that they will include common polymorphisms. The two sets of fragments are tagged in different ways such that homohybrid molecules with identical tags are targeted for subsequent elimination, but heterohybrids, with differentially tagged strands, are conserved. After tagging, the DNAs are mixed, denatured and reannealed to form hybrid fragments of different types as shown in Figure 1. Strands from one individual may reanneal with strands from the same individual to generate self-self hybrids (as isohybrids or as mixed parental hybrids), and with strands from the other individual to produce heterohybrids, either with strands of different parental origin (M/P heterohybrids) or with both strands derived from the same parent (M/M or P/P heterohybrids). Any hybrid with strands from different ancestral chromosomes, hence bearing mispairs at polymorphic sites, is a target for subsequent enzymatic attack by MutS, MutL and MutH (LSHase) and exo III. The DNA that survives the procedures that eliminate self-self hybrids and mismatch specific enzymatic digestion is enriched for the perfectly paired IBD DNA.

### Necessity for high efficiency of removal of non-specific DNA

As shown in Table 1, for any relative pair whose ancestral chromosomes are not related, at least 75% of the DNA mass is targeted for removal. With sibling pairs, for a given locus, depending on the sharing status, the process aims to eliminate 3/4, 7/8 or all of the DNA fragments from the locus. This represents a major challenge because systematic inefficient removal of unwanted DNA reduces the sensitivity of detection of IBD regions. This is because for any of the procedures used for IBD mapping that measure the mass of DNA at specific sets of sequence positions, such as those represented on BAC clone microarrays, a positive signal at a given position indicating IBD enrichment for some pairs must be compared to the signal for pairs where there is no sharing at the position. Previous reports have not addressed a quantitative definition of this problem.

**Table 1:** Partition of hybrid fragments after random reannealing of sibling pair DNAs.

	Heterohybrid			Homohybrid
	Maternal or Paternal		Maternal/Paternal	
	IBD	Non-Identical		

All loci	1/8	1/8	1/4	1/2
Biparental IBD locus	1/4	0	1/4	1/2
Monoparental IBD locus	1/8	1/8	1/4	1/2
Non-identical locus	0	1/4	1/4	1/2

Variation in the distribution of reannealed DNA mass depending on the class of locus sharing. The mass distribution for a monoparental IBD locus is the same as that expected for annealing of grandparent and grandchild DNAs.

Therefore we determined the level of efficiency required to adequately detect IBD enrichment. We express the sensitivity of detecting IBD sharing as a Discrimination Factor (DF), dependent on the overall efficiency of the removal of homohybrid and mismatched fragments. For an expected IBD mass fraction, (I), and a residual surviving fraction of the DNA that was targeted for removal, r, with r = 1 - overall efficiency,

(1)

**Figure 2:**
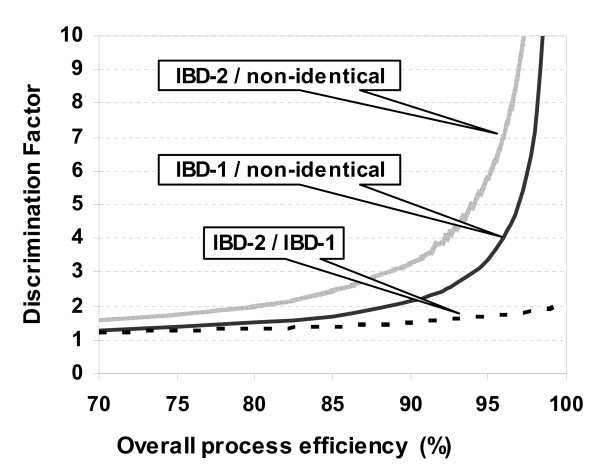
**Discrimination between IBD loci and loci with no sharing as a function of overall process efficiency**. The Discrimination Factor (DF) is the ratio of a value measured when the signal is derived from pairs when the fragments are enriched for IBD sharing to the value for pairs with no sharing. The overall process efficiency indicates the extent that the process has eliminated homohybrids and non-identical heterohybrids. Plots of DF as a function of the efficiency (see Equation 1) are shown for biparental sharing (IBD-2, gray) and monoparental sharing (IBD-1, black). The ratio of the DFs for IBD-2 versus IBD-1 is also shown (dashed).

The behaviour of this function (Figure 2) for the different classes of DNA fragments (Table 1) shows that the overall process efficiency for loci with monoparental IBD sharing must be at least 90% to achieve a minimum of about two-fold discrimination, a reasonable goal for microarray analysis or for real time quantitative PCR. This level of overall efficiency mandates much higher efficiencies for the individual sequential steps of the process. Therefore, we designed various assays, each specific for a different step, to test and select optimum reaction parameters. Some of the same assays are also routinely performed both as periodic quality controls to monitor reagents and enzyme specific activities and as internal and parallel controls during batch processing of multiple DNA sample pairs.

### Robust enrichment of IBD DNA

To produce adequate yields of reannealed fragments, we ensured that all restriction digested gDNAs had a consistent range of fragment sizes, indicating intact gDNA. We optimized several components of the process, notably the choice and concentration of reagents for reannealing in a formamide emulsion [14,30,31] and conditioning of the resin that eliminates fragments resulting from nucleolytic digestion. Quality control during the process also includes monitoring of the DNA concentrations after digestion by the fragmenting restriction endonuclease, after reannealing, after the final enrichment process and after generic amplification to detect any atypical losses or excess yields. Several steps require DNA purification and/or concentration and with a view to automation and robust processing, we perform the process entirely in microtiter plates using ultrafiltration instead of alcohol precipitation. The number of steps has been significantly reduced.

**Figure 3:**
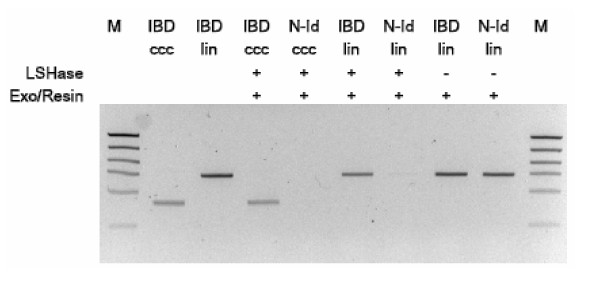
**Evaluation and quality control of LSHase activity**. Substrates of 3.1 kb that mimic reannealed hybrid restriction fragments, either perfectly complementary (IBD) or with a single base pair mismatch (N-Id), and either as covalently closed circular (ccc) or linear (lin) forms, were incubated with LSHase and/or exo III (exo) and treated with the DAP procedure (resin), as indicated. Agarose gels of reaction products were stained with ethidium bromide. Lane M – high mass ladder, Invitrogen.

Using mismatched DNA substrates and perfectly matched controls, we found protein concentrations and reaction conditions that would eliminate all measurable mismatched DNA. We designed model mismatched DNA substrates with a GATC density typical for the human genome (about 1 per 0.5 kb), but with only one mismatch per 3 kb, to ensure that the enzymatic activities that remove non-identical DNA were efficient even for genomic DNA fragments containing a lower than typical density of SNPs. As shown in Figure 3, elimination of mismatched substrates, linear or circular, is dependent on both the nuclease activity of LSHase and subsequent treatment with exonuclease and a resin that removes the digested DNA. We titrated the LSHase activity and the exonuclease/resin procedure such that a significant fraction of the control perfectly paired DNA was also degraded. We also designed two model substrates as internal quality controls in each sample. One substrate is a 4 kb duplex with a single mismatch (MM), and the second is perfectly matched (PM), but with an additional 1 kb of inserted sequence. To each hybrid DNA sample, we added amounts of MM and PM that mimicked single copy gDNA equivalents. Subsequent evaluation by quantitative PCR using primers specific for each substrate ensured that in each sib pair sample all enzymatic functions were sufficiently active to provide adequate discrimination (data not shown). We also included the MM and PM substrates as parallel controls at concentrations sufficient to observe the elimination of the mismatched DNA and conservation of the control DNA (data not shown).

### Discrimination efficiency evaluated by quantitative PCR

**Figure 4:**
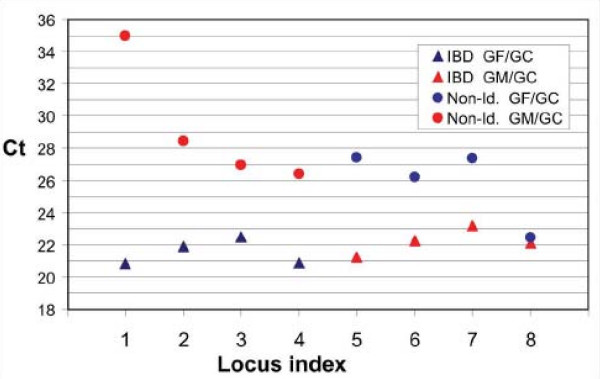
**Quantitative PCR detects an extensive range of enrichment of IBD DNA**. Eight CA microsatellite sites were assayed by qPCR using a universal (CA)_n_-specific probe and IBD-enriched DNA from complementary grandparent-grandchild pairs. Loci 1–4 represent shared loci for the grandfather-grandchild pair and loci 5–8 represent shared loci for the grandmother-grandchild pair. C_t _is the cycle number at which a common threshold level of amplification was achieved.

To determine the extent of discrimination achieved by the IBD enrichment process, we performed quantitative PCR (qPCR) on generically amplified IBD-enriched DNA from CEPH family grandfather-grandchild and grandmother-grandchild pairs. Meiotic recombination points in CEPH family pedigrees have been extensively documented [32,33] and so provide a high resolution map of expected IBD regions between related pairs of individuals. Processing CEPH grandparent-grandchild pairs mimics sib pair analysis in that reannealing produces the same expected mass distribution of hybrid fragments as for monoparental IBD loci sharing between sib pairs (Table 1). As a result of parental meiotic crossing over, the sharing status of each region for a grandfather-grandchild pair is always the opposite to that of the grandmother-grandchild pair. Figure 4 shows that IBD loci can be distinguished from non-identical loci over a range of copy number concentrations. Differences in C_t _values of up to 4 to 6 cycles between IBD DNA and non-identical depleted DNA from the two complementary relative pairs indicate Discrimination Factors ranging from about 16 to 64. Therefore for some fragments, there remains less than 1% of the DNA that is expected to be depleted by the process (Equation (1), Figure 2). Based on results from such qPCR assays, we conclude that the IBD enrichment procedure is highly efficient and that generic amplification of the IBD DNA adequately conserves copy number differences.

### Identification of IBD regions for grandparent-grandchild pairs by mapping on BAC microarrays

**Figure 5:**
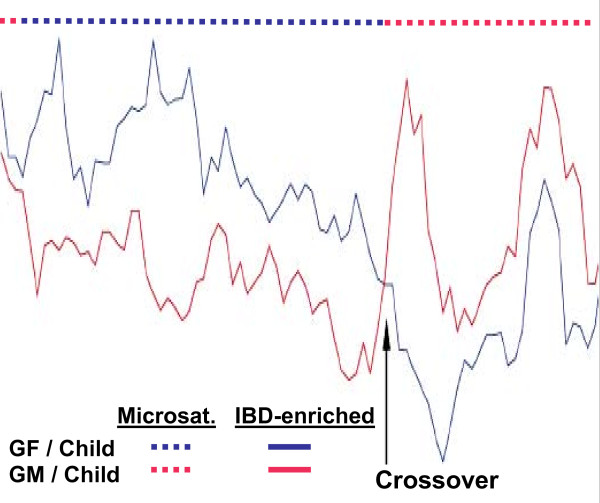
**Confirmation of known meiotic crossovers**. The IBD enrichment process was applied to CEPH family grandparent-grandchild pairs. Amplification, labelling, hybridization and data analysis were performed as described in Methods, except standardization was by division of the normalized ratios by the standard deviation of the ratios of all clones on each array. For chromosome 1, the ratio values for the IBD-enriched DNA (solid lines) are compared with the regions where microsatellite genotypes are shared between a grandparent and grandchild (dotted lines); grandfather (GF)/child, blue and grandmother (GM)/child, red. The arrow indicates a meiotic recombination crossover region.

To map all IBD regions, amplified reannealed hybrid DNA, labelled with Cy3, and amplified IBD-enriched DNA, labelled with Cy5, were hybridized to genome-wide BAC microarrays [34,35]. As described in Methods, after initial filtering, we calculated ratios of the Cy5-labeled IBD-enriched DNA signals to the Cy3-labeled reannealed DNA signals, and normalized the ratios. We standardized the variance between arrays using the variance of all the ratios on each array. Figure 5 shows the profiles of the ratios for complementary grandparent-grandchild pairs. The performance of an immobilized DNA clone is validated when its ratio in an IBD-enriched region is significantly greater than its ratio when the region represents non-identical DNA. Regions of sequential clones with ratios that consistently discriminate the sharing status between the two pairs correspond to regions where the grandparent and grandchild share common microsatellite alleles due to the child's inheritance of the region from the grandparent. Therefore, parental meiotic crossover loci identified by microarray mapping of IBD-enriched DNA are the same as those identified by microsatellite genotyping.

Such experiments with pairs of known IBD status validated the enrichment process and the behaviour of immobilized DNA clones. However, due to uneven distribution of SNPs and different amounts of unique and efficiently annealable sequence, each clone displays its particular characteristics, and hence there is a wide variability in the ratio values between clones. Indeed, as shown in Figure 5, the ratios for some clones detecting enrichment are similar to those of other clones detecting depletion. Therefore, as described in Methods, in subsequent experiments to discover unknown IBD regions, we standardized the ratios by mean centering using the variance of the ratios of each clone obtained from experiments with 150 CEPH sib pairs.

### Application of IBD enrichment and mapping to identify monogenic disease loci

**Figure 6:**
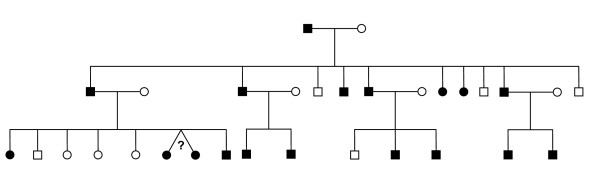
**Osteogenesis imperfecta, an autosomal dominant disease**. Pedigree of Amish family A (Coriell) showing individuals with osteogenesis imperfecta as filled symbols.

Osteogenesis imperfecta patients from four Old Order Amish families, descended from a single founding couple, have a single Gly610Cys mutation in COL1A2, and yet have variable clinical expressivity of the disease. A sample family pedigree is shown in Figure 6. DNA was available for 24 patients and we analyzed 42 affected pairs as shown in Table 2.

**Table 2:** Osteogenesis imperfecta relative pairs

Type of pair	Number of pairs	Expected shared IBD fraction
Sibling	19	0.75
Avuncular	20	0.5
Cousins	3	0.25

**Figure 7:**
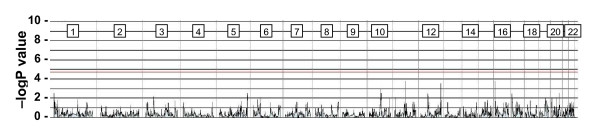
**Linkage test results for CEPH control population**. Genome-wide IBD sharing with standardized ratios was determined as described in Methods. Based on sib pairs from 54 independent families, no clones in chromosomes 1 through 22 surpass the threshold of significance at 2 × 10^-5 ^(red line).

In parallel with the affected pairs, 54 independent control sib-pairs from the CEPH family collection were processed. No linkage is expected at any locus in this collection and as shown in Figure 7, none of the clones showed significant evidence for linkage at the nominal p-value < 2 × 10^-5^. Thus the protocol is not inherently prone to generate false positive results.

**Figure 8:**
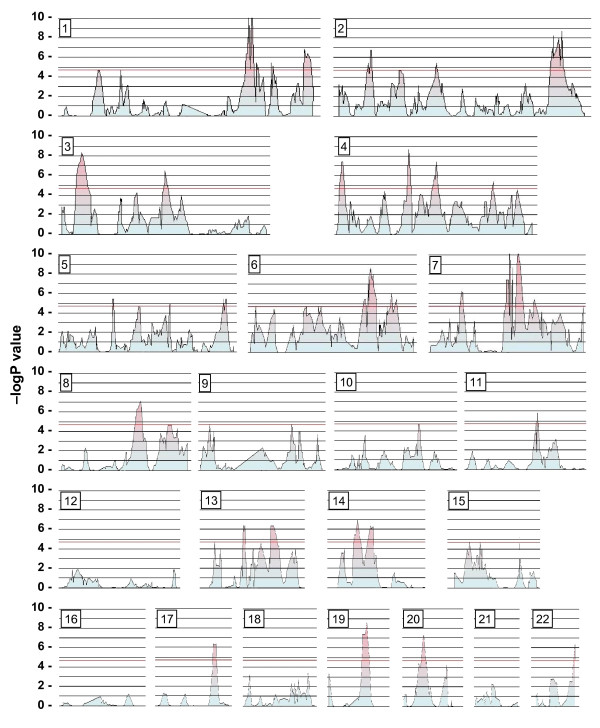
**Sharing of IBD regions among individuals from Amish osteogenesis imperfecta families**. Genome-wide IBD sharing with standardized ratios was determined as described in Methods.

Analysis of the OI family pairs revealed more than 30 peaks of increased IBD sharing with nominal p-values < 2 × 10^-5 ^(Figure 8). However, the analysis of non-independent pairs from a small number of families descended from a common founder will increase the observed IBD sharing between individuals. Therefore, in the context of a monogenic disease the genome-wide threshold for significant linkage is not useful. Some peaks may be linked to specific traits that segregated only within a single family. We therefore considered that the loci more likely to include any cosegregating genes would be represented by peaks with clones showing p-values <10^-6^, for which all pair-wise tests were informative and furthermore where all clones showed IBD sharing. By these criteria, there were two prominent loci on chromosomes 1 and 7 (Figure 8). The chromosome 1 locus spanned about 6 Mb (180.3 to 186.2 Mb) and although this may represent a chance sharing of IBD within these families associated with some trait, it is possible that such a locus harbours a gene that is relevant to the expression of OI. The chromosome 7 locus, (76.4 to 96.1 Mb) includes Col1A2 at 93.9 Mb. As this is the gene bearing the OI causative mutation, we conclude that the IBD enrichment and mapping procedures have successfully identified the disease locus in these families.

## Discussion

Here we have described several critical modifications to a physical positional cloning process to ensure its reliability and to reduce noise in the final mapping analysis. We have recognized a major challenge in applying this methodology, namely that the procedure must eliminate a large fraction of the reannealed DNA, mainly comprised of strands derived from different chromosomes and from potentially confounding self-self isohybrid fragments. We have formally defined the dependence of the discrimination of IBD DNA from non-identical DNA as a function of the efficiency of the process of IBD selection. Therefore, we designed the procedure to ensure highly efficient and specific intermediate yields, and included step-specific quality control assays. Improvements included a reduction in the number of steps, optimization of reannealing, and conditioning of a DNA binding resin to render this key reagent suitably reliable.

The essential component for IBD selection is the enzymatic activity that recognizes and hydrolyzes mismatched DNA fragments. We cloned the three proteins into high yield overexpression vectors, and with various methods, including surface plasmon resonance studies, characterized their activities and optimized storage and reaction conditions [36-38]. We over-titrated the enzymatic activities and other reagents that eliminate unwanted DNA to provide sufficient discrimination for mapping of IBD regions.

Because the yield of the IBD enrichment process is low, we validated a generic amplification method that produced sufficient DNA for mapping. Deviations in copy number representation introduced by the amplification method are not detrimental to the final array ratio determinations because increases or decreases in copy number of different fragments are roughly balanced throughout the long BAC clone sequences and hence in contiguous clones representing an IBD region. Any clone-specific or sequence-dependent deviations are expected to be of similar direction and magnitude in both sample and reference DNAs. The range of discrimination factors between 1.3 and 4 that we observed by BAC clone hybridization might be interpreted as representing the efficiency of the enrichment process. However, microarray analysis is subject to numerous perturbing factors [39] resulting in dynamic range dampening. To exclude any microarray-related factors, we assessed discrimination by qPCR and showed that the process can enrich some IBD fragments at least 10- to 50-fold. Even though numerous hybrid fragments might escape the steps designed to eliminate non-identical DNA and so contribute to noise, the improvements we have introduced to the overall process ensure that the preponderant hybridization signal from the BAC clones is due to strongly enriched IBD fragments. We have also developed a related protocol, Genome Hybrid Identity Profiling, which incorporates these improvements and the use of self-self hybrid discrimination via ligation of oligonucleotide tags; this method enables initial fragmentation of the gDNA with any restriction enzyme that generates efficiently ligatable termini.

The distinction between Mendelian monogenic disease and complex polygenic disease has blurred in recent years [40]; modifier genes may influence the clinical phenotypes of monogenic conditions [41]. To identify candidate loci harbouring disease or modifier genes, extended pedigrees are particularly useful, as any IBD regions conserved in most or all of the afflicted individuals are large, whereas the probability of finding any IBD loci shared among many distant relatives is small [42,43]. To demonstrate adequate performance for genome-wide disease gene mapping, we applied the IBD-enrichment process to Old Order Amish OI family pairs from an extended pedigree and successfully identified a chromosome 7 locus containing COL1A2, the gene bearing the mutation responsible for OI in these families. Another IBD-enriched locus, mapping to chromosome 1q, includes PTGS2 (COX-2), located at 184.9 Mb. COX-2 is expressed in a regulated manner in osteoblasts and is a key regulator in bone formation, interacting with various key proteins of bone metabolism. Variants of COX-2 might affect the clinical outcome of collagen mutations and so may be involved in some of the pleiotropic phenotypes of OI [23,44-46]. The phenotype of mice homozygous for the Col1A2 Gly610Cys mutation mimics the milder clinical expression of the mutation in the Amish families, and the phenotype of heterozygous mice is inconsistent with autosomal dominance [47]. As phenotypic severity of the mutated mice varies substantially depending on the genetic background [48], these mice would be appropriate for constructing additional mutations in candidate modifier genes, such as COX-2.

The potential of family-base studies to identify complex disease genes has yet to be realized; numerous reports have identified linkage peaks of only suggestive p-values and loci are often not replicated [49]. Due to modest gene effects, loci for complex diseases will be difficult to identify unless at least 1000 families are genotyped [50]. Therefore, much effort has been invested in the genome wide association approach using high density SNP genotyping arrays, and many credible associations have been described in case-control studies [51]. Nevertheless, in contrast to linkage analysis, failure to find significant association in a region, even with high density arrays, cannot exclude a disease-related gene in the region [50]. Genotyping several hundred thousand markers generates thousands of significant associations [52], such that the most significant p-values most likely correspond to gene regions unrelated to the disease; whereas the rare true associations are likely to be among the lower ranking p-values of the significant associations [53]. Hence linkage studies that whittle the genome to a few regions, followed by association studies limited to these regions, decrease the multiple testing burden and the likelihood of false associations.

Genotypes from high density SNP arrays are also useful for IBD detection by comparing data from two or more related individuals to find long runs that contain no genotypes inconsistent with common inheritance from the same ancestral chromosome, and that exceed some calculated or simulated cut-off length, thus confidently excluding runs of random IBS [42,43,54]. This approach has been applied with small pedigrees to confirm a previously identified locus for prostate cancer [42], and to identify candidate loci for kidney cancer [54]. This novel means to exploit SNP data, together with the physical method we present here, provide alternative or complementary strategies for gene hunts in family collections and pedigrees. By addressing both the set of variants represented on SNP arrays and additional variants, such as those typically revealed only by fine mapping of regions of associated haplotypes, the physical method may detect shorter IBD regions than the genotyping approach, including regions that might be excluded as likely IBS, or regions of genuine IBD runs broken by erroneous genotypes.

To identify small IBD regions, the resolution of the mapping method must approach the expected mapping resolution of the physical IBD enrichment procedure. In addition, genome-wide mapping of IBD-enriched DNA should preserve the high level of relative enrichment revealed by qPCR. We expect that next-generation resequencing methods [55] will permit mapping IBD regions by scoring sequence read depth. Analysis, including genotyping of sequenced SNPs, would be restricted to unique sequences and restriction fragments with some minimum density of SNPs of high minor allele frequencies, thus ignoring the sequences that contribute to dampening of discrimination in microarray analysis. In addition, sequence selection based on resequencing performance with pools of IBD-enriched DNA from CEPH family pairs would generate a genome-wide set of sequences that accurately report IBD sharing. Considering that reannealing to generate unique sequence hybrids reduces the genomic representation at least five-fold and that the level of IBD enrichment for well-behaved sequences is 10- to 50-fold, very few sequencing runs would be sufficient to obtain a depth of coverage comparable to the depth that permitted calling heterozygous SNPs after resequencing an entire human genome, requiring about 70 runs [56]. Sufficiently deep coverage may permit distinction between monoparental and biparental sharing in IBD-enriched regions.

## Conclusion

We have established a robust process that physically selects and maps genomic regions that are shared between family members. The methodological approach, originally proposed by Ford and colleagues to isolate "inheritance units" [14,15], is now practicable for the simultaneous processing of several hundred relative pairs such as sibling pairs under rigorous quality control conditions. The improved process enabled mapping of loci for a monogenic trait, osteogenesis imperfecta. Using the physical enrichment process, we have previously reported the identification of loci for autism, including a locus on chromosome 16p. With subsequent high density genotyping in this locus, we found that PRKCB1, protein kinase beta, is associated with autism [57]. Thus the procedure is now suitable for enriching for IBD DNA in applications ranging from monogenic to complex diseases. Coupled with mature deep resequencing methods to map the IBD-enriched DNA, the technology will enable increased discrimination that will be required to analyze more distantly related individuals and cost-efficient pools of IBD-enriched DNA samples.

## Methods

### Reagents and DNA

Reagents and suppliers included: restriction endonucleases, Dam methylase and exonuclease III, NE Biolabs; TempliPhi kit, Amersham; Repli-G WGA kit, Molecular Staging/Qiagen; SYBR Green, SYBR Gold and PicoGreen, Molecular Probes/InvitroGen; GenomeHIP reagent components including HyFast gDNA reannealing reagent (HyF), DNA affinity polymer (DAP, a reconditioned derivative of benzoyl-naphthoyl-DEAE cellulose, Sigma), DAP buffer, LSHase (a formulation of E. coli MutS, MutL and MutH), enzyme buffer (EB), a formamide-based array hybridization buffer (AHB), coverslip removal buffer (CRB) and stringent wash buffer (SWB), IntegraGen; Multiscreen filtration plates, Millipore; QIAquick glass-fibre purification plate, Qiagen; UltraGAPS slides, Corning.

CEPH family genomic DNAs were either prepared from immortalized tissue culture cells with the Recoverease kit (Stratagene), a procedure that yields highly intact dialyzed DNA [58], or obtained from the Coriell repository. BAC clones were obtained from InvitroGen or from the Central National de Séquencage, Genoscope (Evry, France). Coriell provides DNA from osteogenesis imperfecta families A, B, C and D, (for pedigrees and phenotypes, see ).

### DNA quantification

We measured DNA concentrations with PicoGreen in a 384-well fluorescent plate reader using calf thymus DNA as standard.

### Hybrid reannealed DNA

We digested genomic DNA (gDNA) from pairs of relatives with Pst I and purified and concentrated the digests with Multiscreen Manu-30 ultrafiltration. We quantified the purified, digested DNA and verified the expected range of fragment sizes by agarose electrophoresis. We used Dam methylase to tag one of the Pst I-digested gDNAs. We combined 1.5 μg of each of the samples, denatured the DNA by incubation in 0.15 M NaOH at RT for 10 min, and neutralized the solution by addition of HyF buffer and phenol. We reannealed complementary strands by shaking the emulsion for 18 hours and recovered the aqueous phase after mixing with chloroform. We immobilized duplex fragments on glass fibre (QIAquick) in the presence of a chaotropic salt to eliminate non-renatured single stranded DNA [59]. We eluted the hybrid renatured fragments with TE and removed an aliquot for labelling and hybridization to microarrays.

### Enrichment for IBD DNA

We incubated the reannealed hybrid DNA with LSHase in EB at 37°C for 15 min and heated for 10 min at 65°C. We incubated the product of the LSHase reaction with Mbo I and Dpn I for 30 min at 37°C and 10 min at 65°C. Then, we added exo III, incubated for 30 min at 37°C, added DAP buffer and treated with DAP to isolate IBD-enriched duplex DNA free of single-stranded digestion products.

### Amplification of IBD-enriched DNA

To amplify the IBD-enriched DNA, we used either TempliPhi or Repli-G, as recommended by the manufacturers with minor modifications. We denatured the reannealed hybrid DNA or the IBD-enriched DNA with NaOH and amplified for 16 hrs at 30°C. We purified the amplified products by ultrafiltration and quantified the DNA. The extent of amplification ranged from about 250- to 2000-fold, or the equivalent of about 8 to 11 doublings.

### Array design

With the aim of choosing 3000 clones with 1 Mb spacing, we used the program CloneTrek (IntegraGen). Clone Trek's input includes essential features of each clone and two parameters, the distance between two clones on the tiling path and the minimal distance accepted between two clones. Initially, all clones in the NCBI clone registry are placed on a tiling path. Clone Trek's iterative algorithm: for each chromosome, while there are clones whose removal would create acceptable gaps, identify and remove the least favoured clone. The least favoured clone has the lowest score with respect to various defined criteria, including FISH data, STS content, sequencing status, size and mapping status. The arrays had 4 replicates of 2779 clones, including 2266 mapped to a unique genomic position with the Build 36 assembly (May, 2006). Average spacing was 1.2 Mb and median spacing 0.95 Mb. Duplicate sets of blocks were printed in two zones in order to maximally separate the two sets of duplicates printed in each block. Various controls including 15 rice BACs were also printed in quadruplicate. Subsequent versions of the arrays have 5500 clones.

### Array printing

We purified BAC DNA using alkaline lysis, filtration and isopropanol precipitation. We amplified BAC DNA as described above for amplification of IBD-enriched DNA. Clone identity was verified by end sequencing of all clones and restriction digest fingerprinting of some clones. We checked the fidelity of amplification by verifying that sample BAC fingerprint patterns matched before and after amplification. We digested 4 to 20 μg of each amplified BAC with Alu I and purified the digests by ultrafiltration. The DNA was dried by vacuum centrifugation and resuspended in 12 μl of 50% DMSO. We printed the DNA on GAPS2 slides (Corning) with a BioRobotics MicroGridII arrayer using BioRobotics quill pins; spot diameters were about 100 microns. We irradiated arrays with 100 mJ of 254 nm UV light and then baked them at 80°C for two hours. We scanned all slides and examined the images of auto-fluorescence to identify any pin-specific problems. We validated the printing batches of about 100 slides by SYBR green staining and hybridization tests on selected slides.

### Mapping of IBD-enriched DNA on microarrays

Amplified reannealed hybrid DNA, labelled with Cy3, and amplified IBD-enriched DNA, labelled with Cy5, were hybridized to the BAC microarrays to enable a ratiometric analysis [34,35]. Labelling reactions of 30 μl were for 16 to 18 hours at 37°C with Klenow (exo-) DNA polymerase (NE Biolabs) and contained 1 μg of DNA, 125 μM random octamers, 120 μM dATP, dGTP, TTP, 60 μM dCTP and either 50 μM Cy5-dCTP or 50 μM Cy3-dCTP. We purified the labelled DNA by spun gel filtration through Sephadex G50 (APB Biotech) in HV45 microplates (Millipore). The specific fluorescence (fluorochromes/Kbp, fl/Kb), of each probe, as determined by DNA quantification and Cy-specific fluorescence readings in a fluorescence plate reader using Cy-dCTPs as standards, ranged from 20 to 50 fl/Kb. We prepared hybridization mixes by mixing Cy5- and Cy3-labeled DNA, concentrating by vacuum centrifugation and resuspending in 35 μl of AHB containing Cot1 DNA. Array slides were blocked by incubation in 10% BSA, 0.01% SDS at 37°C for 30 min. We pre-hybridized slides with 40 μl AHB containing 730 mg/ml salmon sperm DNA at RT for 30 min. We removed about 30 μl of the prehybridization mix, deposited the hybridization mixes on the arrays, covered with Hybrislips (Grace) and placed the arrays in individual hybridization chambers (Corning) in a water bath at 42°C for 2 to 3 days. We removed coverslips by gentle agitation in CRB, rinsed in 2× SSC, soaked in SWB at 45°C for 10 min, and then briefly rinsed slides in a series of baths: 0.2× SSC, filtered 0.1× SSC and 70% isopropanol. We have also tested and validated various commercial labelling kits, hybridization buffers and alternative protocols, including washing at higher temperatures in the absence of formamide.

### Image and microarray data analysis

Arrays were scanned using an Agilent scanner and fluorescent intensities were corrected by subtraction of local background using GenePix^® ^Pro 5.1. Spots with fluorescent signals indicating partial saturation (> 50,000) or signals less than 2 times the mean of the backgrounds from all autosomal clones were excluded. A ratio value of IBD-enriched DNA versus reannealed DNA was determined based on the four spot replicates for each clone. Clones with less than three morphologically acceptable replicates or with excessive variance of replicate ratios (Var(replicate) - Mean(Var(replicates)) > 2 * Var(Var(replicates)) were eliminated. Median ratios of the replicates for each clone were computed and data were normalized between arrays by dividing the ratio of each clone by the mean of the ratios of all autosomal clones. Unless otherwise noted, the ratios of each clone were standardized using 150 full sib pair controls by subtracting the mean of the control ratios of the clone and dividing by the variance of the control clone ratios. Only the clones mapped to a unique genomic position were used in the analysis.

### IBD determination and linkage analysis

We determined a moving average (MA) ratio for each clone. With R_k _as the standardized ratio of clone k, d_i _the physical distance weight for neighbouring clone i (d is inversely proportional to the distance in Mb from the clone, using a weighting function based on a normal distribution), and m the number of flanking clones on each side of clone k, then , the MA ratio of clone k is obtained:

(2)

We set m as three, corresponding to a moving window of seven adjacent clones. As we expect that the status of an average of 75% of the clones is IBD, a threshold ratio T was determined such that 75% of the MA-ratios from all clones and all sib pair controls were greater than T. We set the IBD status to one for clones with MA-ratios greater than T and to zero for clones with MA ratios less than T. After these binary IBD scores were determined for each clone for each affected relative pair, we then counted the number of pairs that were IBD at each clone. A region represented by a clone or a series of consecutive clones is linked to the trait only if the number of affected pairs that are IBD for the region exceeds the number of pairs that by chance could have received copies of the same ancestral region. The null distribution of chance sharing appropriate for studies with different types of relative pairs was determined as described [60]; alternative methods to determine the null distribution may be appropriate for various experimental designs, including those with inbred pedigrees [61-64]. To limit the genome-wide probability of false linkage to 5%, we used the P-value of 2 × 10^-5 ^to set the pointwise significance threshold for declaration of significantly increased sharing [65].

### Quantitative PCR

PCR amplifications were quantified by real time qPCR, using a common probe specific for CA microsatellite repeat sequences [66,67]. Reactions of 25 μl contained 5 ng of amplified IBD-enriched DNA, AmpliTaqGold buffer, 0.2 mM each dATP, dCTP, dGTP, TTP, 0.4 mM dUTP, 5 mM MgCl2, 0.005 U/μl uracil-N-DNA glycosylase (Sigma), 0.03 U/μl AmpliTaqGold DNA polymerase (Roche), 0.2 μM each primer and the probe oligonucleotide 5' FAM-(CA)_14_-TAMRA. Incubations were at 37°C for 10 min, 95°C for 10 min, followed by 35 cycles at 95°C for 15 sec and 60°C for 1 min.

## Availability

The microarray gpr files and three annotation files can be obtained from .

The file "Osteogenesis_samples.txt" lists the Coriell osteogenesis imperfecta samples.

The file "Osteogenesis_relative_pairs.txt" lists the experimental pairs of relatives and the identity codes for the microarray gpr files.

The file "BAC_clones.txt" lists the clone names and their chromosomal positions. If it was not possible to confidently position a clone, it is annotated as "Null".

## Authors' contributions

PB wrote the manuscript and, with expert participation by CM, directed the development of the enzymology and technology for IBD enrichment, DNA amplification and microarray printing and hybridization. MV prepared DNA and performed experiments and hybridizations. JPS manufactured the arrays, prepared DNA and performed experiments and hybridizations. FR directed the implementation of the protocol in a production setting. DS guided the qPCR experiments. SR, RF and NC participated in the design of the OI study. PL designed and wrote the CloneTrek program and managed data bases. JH conceived of the study and participated in drafting the manuscript. AP participated in drafting the manuscript and designed and performed the statistical analysis with assistance by FT. All authors read and approved the final manuscript.
